# Methodological considerations on immunoadsorption versus sham treatment for post-COVID syndrome

**DOI:** 10.1016/j.lanepe.2026.101842

**Published:** 2026-09-04

**Authors:** Kamyar Mansori, Mojtaba Soltani-Kermanshahi

**Affiliations:** a Social Determinants of Health Research Center, Health and Metabolic Diseases Research Institute, Zanjan University of Medical Sciences, Zanjan, Iran; b Social Determinants of Health Research Center, School of Medicine, Semnan University of Medical Sciences, Semnan, Iran

We read the paper by Marco Stortz et al.^1^ which is published in the Journal of the Lancet Regional Health - Europe in June 2026 with great interest. The authors aimed to determine whether autoantibody depletion by immunoadsorption is effective to reduce the symptom burden of patients with post-Covid syndrome, with a cross-over study. Although the results of the study are very interesting, however, it seems that several statistical and methodological issues should be considered when interpreting the results.

First, the authors performed a cross-over study. That is an experiment in which subjects are administered first one treatment, then crossover to receive the second and subsequently crossed over to receive the third and so on. One of the factors tending to complicate the analysis and interpretation of the results of a crossover study is the possibility that the effect of the treatment given in one period carries over into the next period and thus obscures the effect of treatment given then. A solution is to impose a washout period during which the patient is weaned from the first treatment.^2^, ^3^, ^4^ Thus, the first critique is to define the washout period in the study. Second, the final results in Marco Stortz et al.^1^ study developed without checking the carryover effects. Grizzel suggested that in the crossover study, the analysis of the data begin with a test of equality of carryover effects^4^^,^^5^ and the last is the normality test of variables. We do not see any results for normality test.

## Contributors

MSK: conceptualisation, methodology, writing—initial draft, editing, revisions, and supervision; KM: investigation, editing, and revisions; Both authors read and approved the final manuscript for submission.

## Declaration of interests

None.

## References

[1] Stortz M., Kommer A., Meineck M. (2026). Immunoadsorption versus sham treatment for Post-COVID syndrome: a randomised sham-controlled crossover trial. Lancet Reg Health Eur.

[2] Fleiss J.L. (2011).

[3] Lui K.J. (2016).

[4] Mansori K., Soltani-Kermanshahi M. (2020). The effects of eugenol nanoemulsion on pain caused by arteriovenous fistula cannulation in hemodialysis patients: a randomized double-blinded controlled cross-over trial. Compl Ther Med.

[5] Grizzle J.E. (1965). The two-period change-over design and its use in clinical trials. Biometrics.

